# Evaluation of a workflow for cone‐beam CT‐guided online adaptive palliative radiotherapy planned using diagnostic CT scans

**DOI:** 10.1002/acm2.13841

**Published:** 2022-12-26

**Authors:** Koen J. Nelissen, Eva Versteijne, Suresh Senan, Daan Hoffmans, Ben J. Slotman, Wilko F. A. R. Verbakel

**Affiliations:** ^1^ Department of Radiation Oncology Amsterdam UMC location Vrije Universiteit Amsterdam Amsterdam The Netherlands; ^2^ Cancer Center Amsterdam Cancer Treatment and Quality of Life Amsterdam The Netherlands

**Keywords:** antropomorphic phantom, metastases, online adaptive radiotherapy, simulation CT free workflow, single visit palliation

## Abstract

**Purpose:**

Single‐visit radiotherapy (RT) is beneficial for patients requiring pain control and can limit interruptions to systemic treatments. However, the requirement for a dedicated planning CT (pCT)‐scan can result in treatment delays. We developed a workflow involving preplanning on available diagnostic CT (dCT) imaging, followed by online plan adaption using a cone‐beam CT (CBCT)‐scan prior to RT‐delivery, in order to account for any changes in anatomy and target position.

**Methods:**

Patients previously treated with palliative RT for bone metastases were selected from our hospital database. Patient dCT‐images were deformed to treatment CBCTs in the Ethos platform (Varian Medical Systems) and a synthetic CT (sCT) generated. Treatment quality was analyzed by comparing a coverage of the *V*95% of the planning/clinical target volume and different organ‐at‐risk (OAR) doses between adapted and initial clinical treatment plans. Doses were recalculated on the CBCT and sCT in a separate treatment planning system. Adapted plan doses were measured on‐couch using an anthropomorphic phantom with a Gafchromic EBT3 dosimetric film and compared to dose calculations.

**Results:**

All adapted treatment plans met the clinical goals for target and OARs and outperformed the original treatment plans calculated on the (daily) sCT. Differences in *V*95% of the target volume coverage between the initial and adapted treatments were <0.2%. Dose recalculations on CBCT and sCT were comparable, and the average gamma pass rate (3%/2 mm) of dosimetric measurements was 98.8%.

**Conclusions:**

Online daily adaptive RT using dCTs instead of a dedicated pCT is feasible using the Ethos platform. This workflow has now been implemented clinically.

## 1 INTRODUCTION

Radiotherapy (RT) is an effective treatment for metastatic disease, with single‐fraction treatments providing pain relief in a majority of these patients.^1^, ^2^, ^3^, ^4^, ^5^ Currently, most palliative treatments utilize a dedicated planning CT (pCT), with additional time required for treatment planning, which results in longer waiting times or multiple visits for patients. Treatment shortly after first consultation at the RT department is desirable for faster pain control and can potentially limit interruptions to planned systemic treatments. Furthermore, it can reduce travel and waiting times, possibly improving patient convenience.^6^, ^7^, ^8^

A workflow using available diagnostic imaging for treatment planning has been clinically implemented for simple 3D conformal plans at some institutions.^9^, ^10^, ^11^ However, any differences in patient positioning, body contour, and tumor size during RT‐delivery could result in undesirable deviations when intensity‐modulated plans (IMRT) are delivered.^10^ IMRT plans are desirable as they can decrease doses to organs at risk (OARs).^12^, ^13^, ^14^ However, previous studies that used diagnostic CTs (dCT) for treatment planning in an urgent palliative RT setting advised caution in more complex cases.^9^, ^10^ Other authors have attempted to replicate positioning during treatment by using an vacuum‐bag, 6D setup corrections, or applying rigid shifts and rotations to account for differences in positioning during treatment.^10^, ^11^, ^15^, ^16^ Overall, these studies suggested that dCT‐based treatments are feasible, but that further investigation of the workflow is required in integrated treatment platforms.

After the clinical introduction of daily online adaptive treatment on the Ethos platform (Varian Medical Systems, Palo Alto, CA, USA), we developed a dCT‐based workflow for palliative RT. The workflow involved initial treatment planning on available dCT, and subsequently on‐couch plan adaption on a cone‐beam CT (CBCT)‐scan during the first clinical visit to account for any changes in target, OAR, and body anatomy. Before implementing this workflow in the prospective FAST‐METS study (NCT05288608), we performed a retrospective study to investigate the feasibility of omitting a dedicated pCT for palliative RT using online adaptive treatment planning.

## 2 METHODS

This retrospective study investigated the feasibility of omitting a dedicated pCT for palliative RT using online adaptive treatment planning on the Ethos platform.

### 2.1 Patient selection criteria

Patients were identified from the departmental information management system (ARIA, Varian Medical Systems) based on the following criteria: (1) received palliative RT for metastases in ribs, spine, or pelvic regions with either a single fraction of 800 cGy or five fractions of 400 cGy, with CBCT setup on the Ethos; (2) had provided written informed consent for the use of their data for retrospective studies; (3) had a dCT available with full body contour (missing lateral body contours of <2 cm were acceptable); and (4) had a dCT performed within 6 weeks before treatment.

### 2.2 Pretreatment planning

For each patient, imaging data consisting of a dCT and a CBCT were anonymized and then imported into the emulator, which is a copy of the clinical Ethos software on a virtual server provided by the manufacturer (Varian Medical systems).

Clinical target volumes (CTV) and OARs were redefined on the dCT by a radiation oncologist, using the original clinical contours as a reference. The planning target volume (PTV) included the CTV plus a 5 mm isotropic margin (and 10 mm for patients 9 and 15). Treatment planning was done using existing departmental Ethos templates for each anatomical target site with prioritized clinical goals (Table 1). Clinical plan templates were available for the following target areas: right‐rib, left‐rib, bilateral‐rib, cervical spine, thoracic spine, lumbar spine, and pelvic region. So‐called influencer structures, which are used in the subsequent structure‐guided deformable registration in the adaptive workflow, were defined for each anatomical target area. The influencer for targets in thoracic spine was the spinal canal; bowel for the lumbar spine and pelvic regions, and both bowel and spinal canal for the sacral spine. Goals on targets were defined with a higher priority (1) compared to the OAR goals (2 or 3). Planning was performed using a full 3 arc volumetric modulated arc therapy (VMAT) or 12‐field (equidistant) IMRT technique. When necessary, normalization (PTV*_V_*_95_ ≥ 95%) or a small deviation in an OAR goal was permitted. All plans were reviewed by a medical physicist. These were defined as the reference plans (TP_R_).

**TABLE 1:** Clinical goals used in the treatment planning system templates

Structure	Clinical goals
CTV	*V*95% ≥ 98%
PTV	*V*95% ≥ 95% *D*99% ≥ 85% *D*_max_ ≤ 115% *D*2% < 110%
Spinal canal + 3 mm	*D*0.1 cm^3^ ≤ 100% (variation to 110% acceptable)
Other OARs (bowel, lungs, kidneys, esophagus)	*V*38% < 20% *V*50% < 10% *V*75% < 5% *D*_mean_ < 25% *D*_max_ < 95%
Body	*D*1 cm^3^ < 107%

Abbreviations: CTV, clinical target volume; OAR, organ at risk; PTV, planning target volume.

### 2.3 Adaptive workflow

**FIGURE 1:**
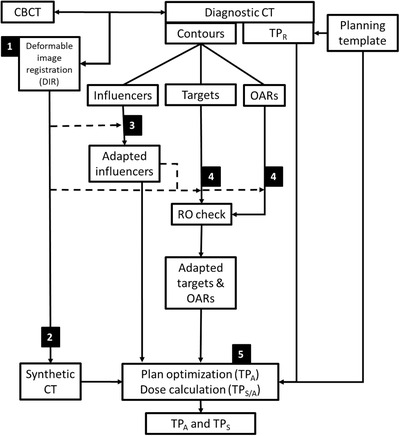
Adaptive workflow schematic. Showing steps 1–5 in the emulated workflow on Ethos, these are referenced in Section 2.3: radiation oncologist (RO), reference treatment plan (TP_R_), adapted treatment plan (TP_A_), scheduled treatment plan (TP_S_), and organs at risk (OARs).

On‐couch treatment in the emulator test environment used clinical CBCT images acquired during the clinical treatment session. Within the Ethos workflow,^17^, ^18^ the dCT is co‐registered to the CBCT using a B‐spline deformation model and Mattes mutual information as cost function (Figure 1, number 1).^19^ The generated vector fields were used to propagate the HU‐value for each voxel of the dCT to the CBCT, thereby creating a synthetic CT (sCT) for use in dose calculations (Figure 1, number 2). Influencers were automatically delineated on the CBCT by Ethos segmentation algorithms, before contours were reviewed and adapted (if required) by the radiation oncologist (Figure 1, number 3). Targets and OARs were automatically propagated using a structure‐guided vector field. These structures were reviewed by the radiation oncologist and adapted if necessary (Figure 1, number 4).^20^, ^21^ After target acceptance, the other OARs are propagated, and dose distributions were calculated for the scheduled treatment plan (TP_S_) on the sCT (Figure 1, number 5). The TP_S_ represents the expected dose without plan adaption on the daily patient model (sCT). Next, the adapted treatment plan (TP_A_) was optimized for the daily anatomy on the sCT, using the same template that had been used for initial treatment planning. The data were exported to the nonclinical Aria (v16.0) database, and differences among TP_S_, TP_R_, and TP_A_ were evaluated for target clinical goals (in *V*95%_CTV_ and *V*95%_PTV_). For the OARs values, the *V*300, *V*400, and *V*600 cGy were determined for the lungs (*n* = 5), esophagus (*n* = 5), bowel bag (*n* = 6), kidneys (*n* = 4), and *D*0.1 cm^3^ for the spinal canal (*n* = 10). Differences were calculated by TP_R_ or TP_S_, minus TP_A_ at dose–volume histogram (DVH) points *V*300, *V*400, *V*600 cGy, and averaged, and the significance of observed average differences was evaluated by using a paired Wilcoxon signed‐rank test in R‐Studio (version 4.1.1).^22^, ^23^

### 2.4 Validation patient model and workflow

#### 2.4.1 Dose recalculations and comparison CBCT and sCT

We considered the sCT as the on‐couch patient model as it was used for plan optimization. Doses were recalculated on both the sCT and CBCT (Eclipse Treatment Planning System 16.01.10, Acuros external beam 16.1.0), where the CBCT represents actual patient anatomy. Adapted structure sets defined by the radiation oncologist during the on‐couch emulation were copied by rigid registration from the sCT to the CBCT, resulting in structures of identical shape and position in relationship to the beam isocenter on both scans. Dose calculations on CBCTs have previously been shown to be an acceptable predictor of dose, with average deviations of 2% compared to a regular CT calculation.^24^ This method was used for the comparison of patient models in the adaptive workflow. For this study, a patient model was considered to be challenging when the CBCT and dCT body contours or internal anatomy (e.g., extension of the lungs) differed, which could lead to errors in dose to targets and OARs. Differences in goals (CTV, PTV, and OARs) between the CBCT and sCT were evaluated.

#### 2.4.2 On‐couch anthropomorphic phantom film measurements

**FIGURE 2:**
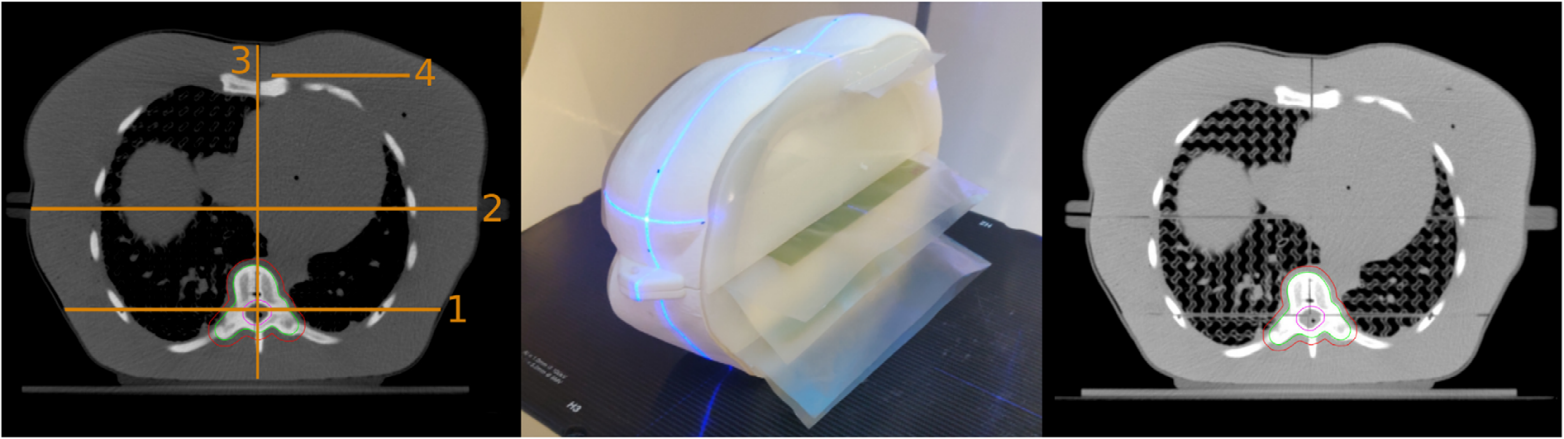
CT images of the anthropomorphic phantom used for film measurements. The left panel shows the possible planes for film insertion, middle panel the phantom on couch with inserted film, and the right shows a CT with low density lung grid visible, with the targets and organs at risk (OARs) delineated.

An anthropomorphic phantom and Gafchromic EBT‐3 film (Ashland Advanced Materials, Bridgewater, USA) were used for on‐couch testing of the workflow and validation of TP_A_. The 3D phantom was printed and developed at the 3D Innovation Lab of the VU University Medical Center.^25^ A modification to the original phantom published in 2017 allowed film measurements inside the phantom (Figure 2) in coronal planes (number 2) through the spine and lungs. Three film measurements were performed on the phantom for three different plan adaptations after completing the on‐couch adaptive workflow. For these measurements, the pCT was made with the phantom rotated compared to the on‐couch positioning: roll/pitch/yaw of: (5,5,0), (5,5,3), and (3,3,3) degrees. In order to get an impression of the total duration for the on‐couch procedure (which could be different on the emulator), this time was measured from the end of the first CBCT to the end of the last CBCT. In total three CBCTs were made, one used in the adaptive procedure, second for position verification, and the third as the final time point. The films were digitized using an Epson Expression 12000XL Pro flatbed scanner in transmission mode (48‐bit, 72 DPI). An in‐house developed Matlab code was used to apply a lateral correction and translated the red‐channel measured intensity to dose using a calibration curve.^26^ OmniPro I'mRT (IBA Dosimetry) was used to calculate the average dose of two films per measurement and to compare it with the dose calculated in Eclipse using a gamma analysis (3% dose difference, 2 mm distance to agreement).

## 3 RESULTS

### 3.1 Patient selection

A total of 37 patients were chronologically identified from the database in a period of 9 months, of which 22 were excluded for reasons, such as a lack of documented informed consent for retrospective studies (*n* = 6), unsuitable CBCT data (*n* = 3), or the lack of a suitable dCT (*n* = 13). This resulted in 15 eligible patients for further analysis, with an average interval of 21 days between dCT and day of treatment. Table 2 summarizes treatment locations and the time between dCT and RT.

**TABLE 2:** Overview of radiotherapy (RT) sessions of patients included: all patients (except denoted with the superscript “b”) were treated with 12‐field intensity‐modulated radiotherapy (IMRT) technique

		Target volume (cm^3^)			Monitor units adapted plan	
#	Target	**TP_R_**	**TP_A_**	Interval dCT to RT (days)	**TP_R_**	**TP_A_**
**1**	Thoracic spine	75	92	13	3966	3368
**2**	Thoracic spine	120	130	25	4210	4173
**3**	Thoracic spine	232	242	13	3074	3546
**4**	Thoracic spine	80	85	19	3843	5807
**5^b^**	Thoracic spine	266	267	1	2016	2077
**6**	Lumbar spine	40	84	13	2866	2446
**7**	Lumbar spine	79	79	20	3562	3421
**8**	Lumbar spine	225	214	17	3579	3289
**9**	Lumbar spine	94	91	4	3128	3185
**10^a,b^**	Sacral spine	399	411	42	3343	3280
**11**	Sacral spine	339	338	10	3767	3704
**12^a,b^**	Right rib	38	195	42	2543	3499
**13**	Right rib	9	8	37	1830	1789
**14**	Right rib	23	22	29	1407	1622
**15**	Right rib	51	67	28	2970	2823

^a^Indicates data from the same patient but different courses and target areas. In this patient, the same dCT was used, but different CBCTs for both locations.

^b^Technique used was two full‐arc VMAT.

### 3.2 Adaptive workflow

Of the 15 eligible patients, major edits of target contours (defined as edits to >4 CT slices) were required for 4 patients, 7 patients required minor edits to target contours (in <4 slices), and 4 patients no adjustments. The adjustments to target contours arose due to errors in the target propagation, and for one (patient 12), because tumor progression was observed on CBCT. For patient 4, the original dCT missed a part of the body due to a limited FOV (Figure 5b), additionally a change occurred in patient anatomy due to positioning of the arms. These two changes led to a major increase in TP_A_ monitor units calculated on the sCT where this part of the body was added based on the CBCT data. Automatic segmented OAR contours were accepted in all except for two cases, where the esophagus was displaced relative to the target, a change that was not automatically corrected by the algorithm.

### 3.3 Dosimetric analysis clinical goals

#### 3.3.1 Target coverage

**FIGURE 3:**
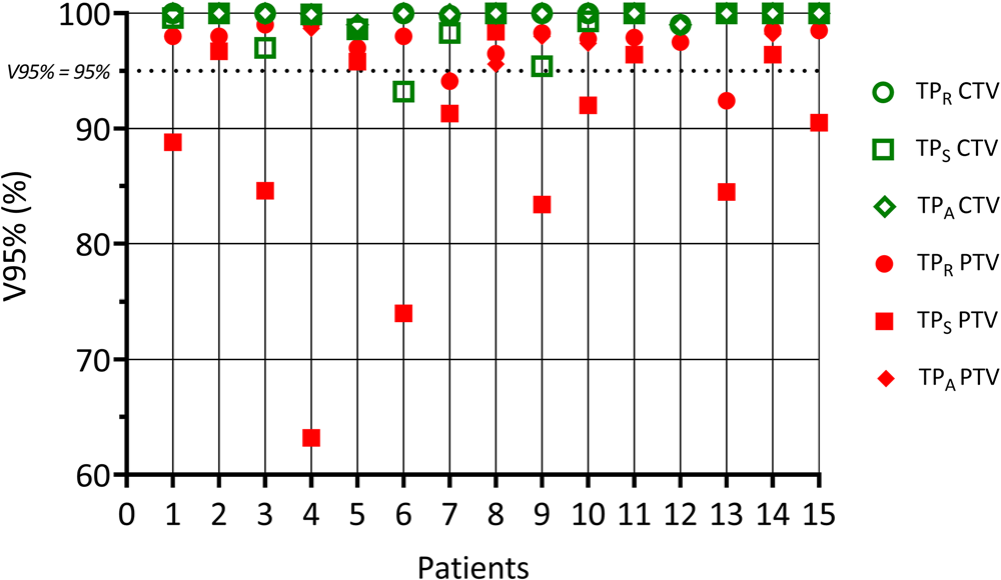
Target coverage comparison among reference (TP_R_), scheduled (TP_S_), and adapted (TP_A_) treatment plans for clinical target volume (CTV) and planning target volume (PTV) *V*95%. Two outliers are not shown in this graph for patient 12: TP_S_ CTV *V*95% = 45.6% and PTV *V*95% = 28.2%.

The target coverages in TP_A_ and TP_R_ were comparable for all patients. Plan target coverages, including TP_S,_ are presented in Figure 3. The mean difference in *V*95% ± standard deviation and *p*‐values between the TP_S_ and TP_A_ target coverage for Δ*V*95%_CTV_ = −4.73 ± 3.40% (*p* = 0.014) and Δ*V*95%_PTV_ = −12.91 ± 4.64% (*p* = 0.001), showing a significant higher percentage of the volume covered by 95% of the prescribed dose for TP_A_. Target coverage of the TP_A_ deviated on average less than 0.2% from TP_R_, with Δ*V*95%_CTV_ = −0.02 ± 0.01% (*p* = 0.37) and Δ*V*95%_PTV_ = 0.19 ± 0.08% (*p* = 0.036), showing an insignificant change in CTV coverage and significant improved coverage of PTV volume. For patient 8, target coverage of the TP_S_ was improved compared to the TP_A_ and TP_R_. This was caused by a change in body anatomy leading to a higher target dose with the original beam setup. The TP_A_ fulfilled the clinical guidelines and lowered OAR *D*_max_ dose.

#### 3.3.2 Dose in OARs

**FIGURE 4:**
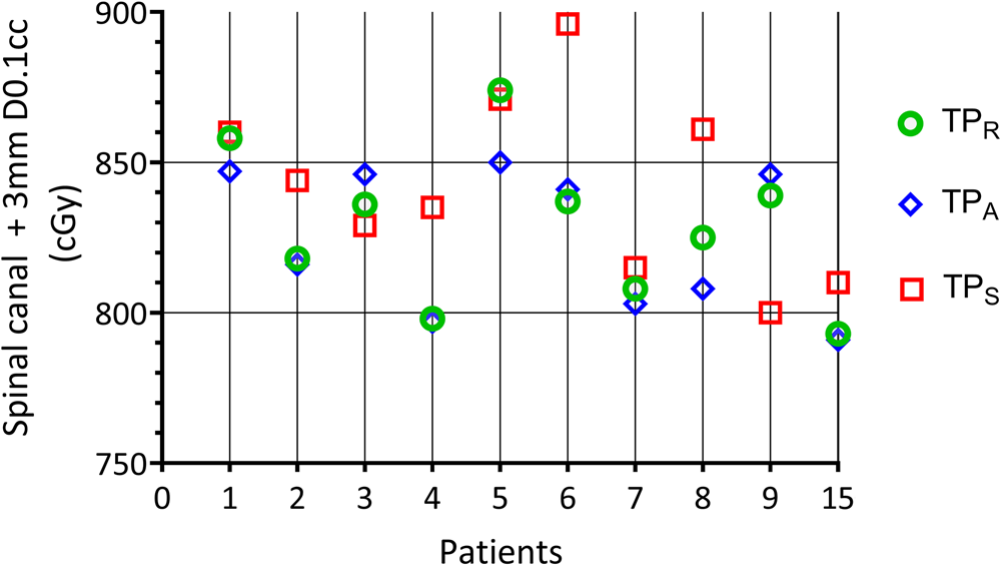
Maximum dose to 0.1 cm^3^ of spinal canal (+margin 3 mm) volume among reference (TP_R_), scheduled (TP_S_), and adapted (TP_A_) treatment plans. For patients 10–14, no spinal canal organ at risk (OAR) was present.

**FIGURE 5:**
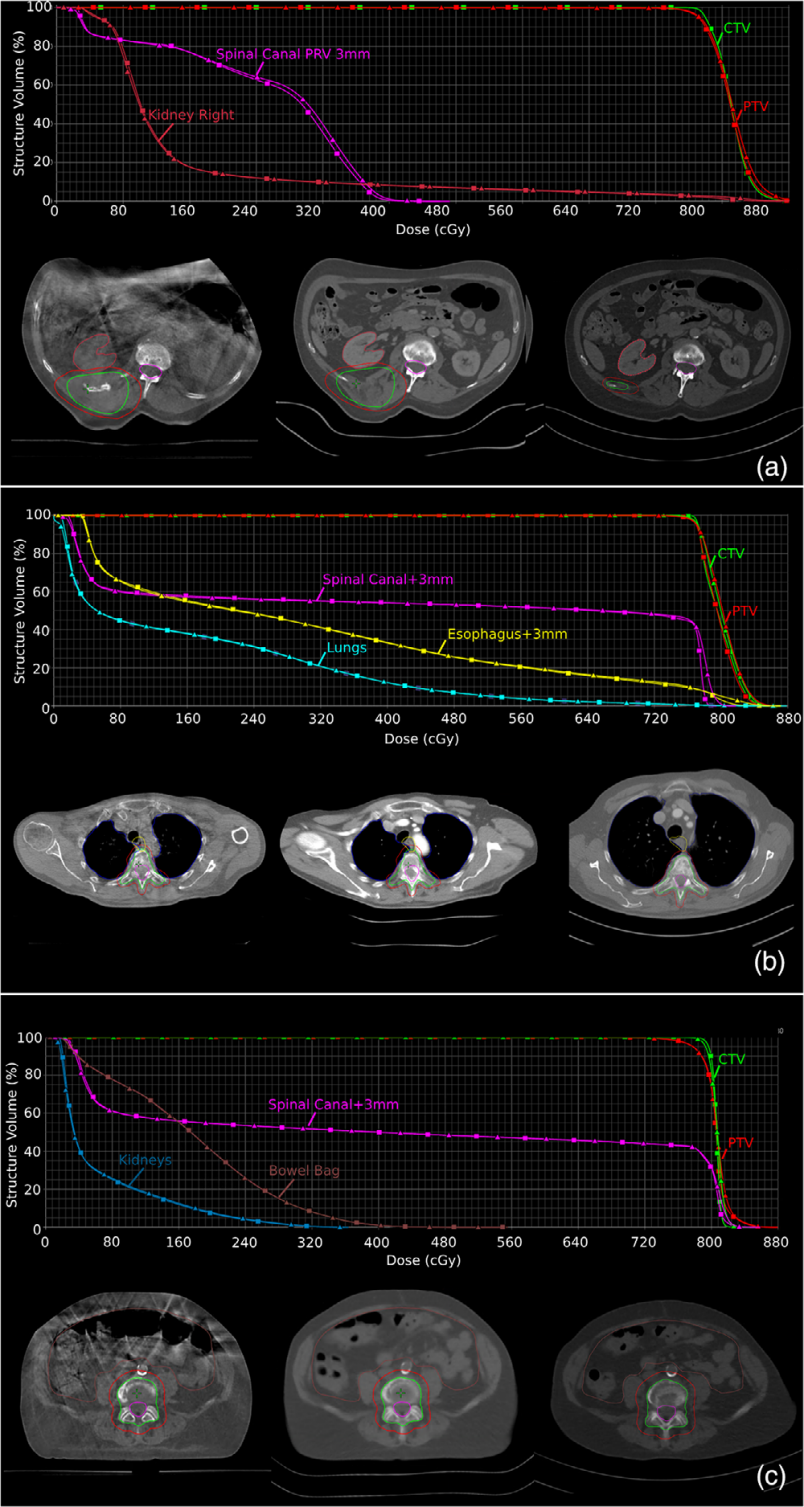
Examples of anatomical differences among the cone‐beam CT, synthetic CT, and diagnostic CT (from left to right, respectively). In addition, for each patient a comparison of the dose–volume histogram between the synthetic CT (square) and the cone‐beam CT (triangle) is shown. Part (a) shows patient 12, (b) patient 4, and (c) patient 9.

Comparison of dose for 0.1 cm^3^ in the spinal canal (including a margin of 3 mm) for TP_A_, TP_S_, and TP_R_ revealed nonsignificant (*p* > 0.1) differences (Figure 4). Dose differences between the TP_R_ and TP_A_ in OARs were insignificant (*p* > 0.2). However, larger differences were observed for OARs when comparing the TP_S_ to TP_A_, with average dose differences in lungs of −0.9% ± 1.4% (*p* = 0.024) and nonsignificant differences in the other OARS (*p* > 0.2, data not shown). In two patients (4 and 12), OARs received a higher dose in the adapted plan. Patient 12 had tumor progression, leading to a larger PTV with increase in dose to the right kidney (Figure 5a). In patient 4, the position of the esophagus differed significantly from the original pCT, leading to a higher esophagus dose in the TP_A_ compared to TP_R_. On average, although not significant (*p* = 0.055), TP_A_ resulted in lower esophagus dose (*V*300, *V*400, and *V*600 cGy) compared to TP_S_ (difference: 6.7% ± 11.1%).

### 3.4 Validation patient model and workflow

#### 3.4.1 Dose comparison on CBCT and sCT

Figure 5 highlights the changes in anatomy for three patients, shown in the dCT, CBCT, and sCT, with a comparison of DVHs calculated on the sCT and CBCT. These three patients were selected based on interesting clinical features, such as changes in body contour, target volume, and densities, in order to provide an indication of some limitations of the proposed workflow. In patient 12, tumor progression led to an on‐couch increase in the target volume of 513% (Figure 5a and Table 2). Patient 4 shows a limited field of view on the dCT and large changes in body contours on‐couch (Figure 5b). Patient 9 (Figure 5c) had a large air cavity in the bowel during the CBCT, which was not found in the sCT for the dose calculations, resulting in differences in the dose distribution between the CBCT and sCT. If a density override was done for this air bubble/cavity, these differences would not have been apparent. Except for patient 9, dose disparities between the sCT and CBCT were minimal (<2% in high‐dose regions).

#### 3.4.2 Verification of workflow using an anthropomorphic phantom and film dosimetry

Testing of the workflow on an anthropomorphic phantom resulted in gamma pass rates (3%/2 mm) of 99.2%, 99.0%, and 98.1% for the three measurements. The entire adaptive on‐couch workflow (CBCT1‐CBCT3) using phantom measurements took 10, 11, and 15 min, including scan matching and target contour adaptation. Workflow was technique dependent, with VMAT optimization time taking up to 6 versus 2 min for 12‐field IMRT.

## 4 DISCUSSION

The main finding of the present study is that a workflow for palliative RT in bone metastases was feasible simulating a single clinic visit when a maximum 6‐week old dCT is used for initial treatment planning, followed by on‐couch plan adaptation based on a CBCT. Specifically, on‐couch plan adaptation was able to account for changes in body contours and target volume, and this approach allowed for the use of highly conformal OAR sparing plans. As high gamma pass rates were observed for the adapted plans consistent with previous work,^27^ we concluded that clinically acceptable treatment plans could be achieved without the use of a dedicated pCT.

Approximately 75% of the patients required the adaptation of target contours by a radiation oncologist. Although this adaptive workflow allowed for contour modifications on the CBCT, this was a time‐consuming step. Adaptation of target volumes also led to longer calculation times as the plan optimization process had to recommence. The Ethos software required optimization times for a 3 arc VMAT of up to 6 min during phantom testing, and additional on‐couch time is not desirable in our patient population. In contrast to the previous work, our study did not encounter issues arising from differences between the curved dCT couch and the flat RT couch.^10^

The observed differences in target coverage between the TP_R_ and TP_A_ were less than 0.2%, a finding comparable to previous studies using 3D conformal plans.^16^ Although dose differences to the OARs varied up to 10% between the TP_A_ and TP_R_, OAR doses generally met plan constraints for palliative treatment. A higher dose to the OARs in TP_A_ seen in two patients arose due to tumor progression and positional variation of the OARs. However, in comparison to current clinical practice, our proposed workflow allows for plan adaptation of the observed changes.

One aspect of this study was the inclusion of only patients with a short time interval (≦6 weeks) between the dCT and RT in order to minimize the likelihood of changes in the target volume. Another technical limitation is that breathing artifacts could impair the quality of the CBCT, leading us to exclude targets that move with respiration. Furthermore, the sCT does not take into account air‐deformations from the CBCT to the sCT. Correcting for this density differences also corrects differences in dose, and the current adaptive workflow does not take into account changes in lower density regions due to, for example, bowel movements. Although a previous study demonstrated that dose differences due to differences in CT HU calibration curves were small, ranging from 1.0%, −0.5%, and −0.2% for lung, liver, and bone.^16^ We postulate that the minor differences of up to 2% observed in our study between dose calculations on the sCT and CBCT are driven by a combination of the CT‐calibration differences,^10^ body contour changes, and small differences in high‐density localization, such as bony tissue. Our study compared doses on the CBCT and sCT in order to check if possible errors in the sCT could lead to clinically significant dose discrepancies. Ideally, this comparison should be done with doses calculated on a pCT‐scan with the patient position being identical to that on CBCT, which unfortunately was not available.

There are a number of areas for further improvement in fast palliative procedures. Some patients may only have magnetic resonance imaging (MRI) scans available instead of a dCT, and research studies on MRI to CT conversion algorithms are awaited.^28^, ^29^ A limitation of the current adaptive software in the Ethos platform is the requirement for a suitable dCT treatment planning, whereas direct contouring and planning on a CBCT‐scan may facilitate faster palliative RT. Finally, similar palliative RT workflows are being evaluated on magnetic resonance linear accelerator systems, with the potential for improved visualization of soft tissue tumors compared to a CBCT‐based workflow.^30^, ^31^ Future work should provide additional insights to determine the strengths and weaknesses of both approaches.

## 5 CONCLUSIONS

In summary, online adaptive RT for metastatic disease can be performed without the need for a dedicated pCT. Clinically acceptable adaptive plans derived using this workflow will enable a combination of consultation and fast treatment within a short interval. This workflow is currently being evaluated in the FAST‐METS trial.

## AUTHOR CONTRIBUTIONS

All authors contributed significantly to the performed work and approved the manuscript for publication.

## CONFLICT OF INTEREST

The department has multiple research collaborations with Varian Medical Systems. This research was funded by a research grant from Varian Medical Systems. Verbakel and Slotman have received honoraria/travel expenses from Varian Medical Systems. Senan has received research funding from Varian Medical Systems and ViewRay Inc.
